# Teething: Is It a Common Cause of Disorder

**Published:** 1887-09

**Authors:** Selwyn A. Russell

**Affiliations:** Albany.


AMERICAN JOURNAL

—OF—

DENTAL SCIENCE.

VOL. XXI. THIRD SERIES—SEPTEMBER, 1887. No. 5.

ARTICLE I.

### TEETHING: IS IT A COMMON CAUSE OF DISORDER.

BY SELWYN A. RUSSELL, M. D., ALBANY.

It is the belief of mothers almost universally that most disorders of children are due to teething; this belief is, very unfortunately, too common among physicians. On the contrary, it is the belief almost unanimously of experienced specialists who devote themselves to the treatment of children's diseases in America and abroad, and whose books are authority with the profession everywhere, that teething is rarely the cause of children's disorders, and, as a rule, the cause of none. The object of my remarks this evening is to show the mistake that is daily made, by the profession as well as the laity, in allowing the so-called teething disorders to go on without treatment till a stage is reached in which the health, and perhaps the life, is in jeopardy. For example, every mother says of her child's diarrhœa that it is a sort of mysterious vent by which is thrown off a mysterious something that would otherwise "go to the brain," and cause disturbance there; and she not only feels sure that the baby's health is protected by this disorder, but is equally sure that without it other more serious disorders would supervene; so she makes no effort to check it. From the sixth month to the twenty-fourth, loosely speaking, the child is cutting its teeth, so that every ailment that occurs during that period will most likely coincide with symptoms of teething. The physician is, of course, anxious to assign a cause to every disorder, and as he knows the mother's prejudice in favor of teething, and being himself averse to careful physical examination of the child, he yields to the mother's bias, says the child is teething, therefore the disorder. How often have serious affections been thus overlooked and the symptoms misinterpreted or altogether ignored!

If a disease is to be considered as a consequence of teething, it must not simply now and then coincide with the cutting of a tooth, but the coincidence should be exact as to time, and repeated, if not with every tooth, at least frequently. We cannot consider any thing the sure cause of an effect so long as another cause is equally probable. As our knowledge of the causes of diseases in children has increased, the importance of teething as a cause has decreased, and we now reject many of the alleged effects of teething that physicians formerly admitted. The chief difficulty arises from ascribing to teething disorders due to other causes, which might have been easily removed at the outset.

If any disorder exist, supposed to be caused by teething, it is inferred that the child is teething, whether it is or not. So the parents are confused, the physician himself being in many instances to blame.

Some disturbances of the digestive system, some nervous disorders, eruptions on the skin, etc., it is known, are occasionally due to teething.

The difference of opinion as to the importance of teething as a cause of disease is not a simple dispute of terms, but one which has a real interest in the nursery. If the parents believe that teething causes all the ailments attributed to it, they are, as is daily seen, likely to regard the ailments as nearly, if not quite as much, a matter of course as the natural teething process, and they consider it useless to try to cure them until teething is complete. As a result of these errors and confusions, it too frequently happens that disorders very tractable at the outset are allowed to progress unopposed until they reach a serious stage. On the contrary, if we assume that teething is rarely the real cause of disease, the parents will seek some other cause for any disturbance that may be present, and endeavor to remove it. (Yale.)

With these few prefatory remarks I beg to read to you quotations from the best authorities on this question, which are taken sometimes from the chapters on dentition, sometimes from chapters on diarrhœa, enteritis, eclampsia, etc. The quotations first given are from authorities who regard teething as a common cause of disorder; following these will be found authorities, as a rule better known, and in the large majority, who look upon teething as a physiological process rarely if ever giving rise to disorders.

Meigs and Pepper, seventh edition, page 412, says: "Dentition may be mentioned as exerting a strong influence in the causation of enterocolitis. That the evolution of the teeth, though a physiological process, is a powerful predisposing cause of diarrhœa and enteritis, cannot be doubted at the present time."

Speaking of cholera infantum, they say: "We believe dentition to be a most powerful predisposing cause of this disease, and yet it is less influential than age, for vital statistics show that it is about twice as fatal in the first year as in the second, though the process of dentition is certainly more active and continuous in the second than in the first year. We have rarely observed it before the beginning of the process of dentition, and it is certainly very rare after its completion."

They also say that it is a frequent cause of laryngismus stridulus. The age at which it occurs most frequently the age from six to eighteen months, the very period during which the process of dentition is most active, would alone go far to show that this must constitute one of the most powerful predisposing, if not exciting, causes.

Day, in "Diseases of Children" (1881), page 69, says: "What share teething exerts in causing diarrhœa it is impossible to say; but there is a close connection existing between these states. When diarrhœa is present we do not hastily attempt to check it if the teeth are piercing the gums, and the mouth is uneasy; still, if the drain continues or is excessive, the child becomes exhausted, and the possibility of convulsions must not be overlooked."

Ellis ("Diseases of Childhood"), London, says on page 10: "During dentition the child's health requires unusual care; the bowels must be regulated, the diet strictly attended to, the gums lanced when they are hot and swollen; the diarrhœa of teething is natural, and if in moderation should not be interfered with."

Vogel ("Diseases of Children," page 109) says: "A mild diarrhœa, five or six passages in twenty-four hours, is very beneficial to teething children, for cerebral affections are thereby most surely prevented. It occurs, in fact, as often as catarrhal stomatitis, and both processes might very appropriately be regarded as physiological conditions, if their aggravations, which often attract attention, did not attain to distinct diseases, and really display serious characters." He thinks the excessive quantity of saliva at this time, swallowed by the child, is the cause of diarrhœa.

Dr. Hillier, London, in his book on "Diseases of Children," says: "Until recently nearly all convulsions of infants were ascribed either to teething or to worms; it is very doubtful whether in a healthy child these causes can produce convulsions at all; in a predisposed subject they no doubt often excite them." (page 380.)

West, in his "Lectures on the Diseases of Infancy and

Childhood," page 507, says: "You will observe that the period of greatest prevalence of diarrhœa coincides exactly with that time during which the process of dentition is going on most actively, and that exactly half of all cases of diarrhœa occured in children between the ages of six months and two years. So close, indeed, is the connection between teething and diarrhœa that a French physician, M. Bouchat, found that only twenty-six out of 138 children entirely escaped its attack during the period of their first dentition, while forty-six suffered from it severely. The older writers on medicine, whose notice this fact did not escape, attributed the disturbance of the bowels to a sort of sympathy between the internal canal and the gums, swollen and irritated by the approach of the teeth to their surface. The frequent observation of cases in which an attack attends the irruption of each fresh tooth, and ceases when it has cut through the gum, shows that such an hypothesis is not altogether without foundation. But, besides the influence of nervous irritation in quickening for a time the peristalic action of the bowels, and thus inducing diarrhœa, it must be borne in mind that there exists during the period of teething a *more abiding cause*, which strongly predisposes to its occurrence. All parts of the digestive canal, and of its dependencies, are now undergoing an active evolution to fit them for the proper assimilation of the varied food on which the child will soon have to subsist. Just as the salivary glands now begin to secrete and pour out saliva in abundance, so the whole glandular system of the intestines assumes a rapidity of growth, and an activity of function, which, under the influence of comparatively slight causes, may pass the just limits of health. In too many instances, causes, fully adequate to excite diarrhœa, are abundantly supplied in the excessive quantity or unsuitable quality of the food which the infant is furnished; for it is forgotten that its condition is one of transition, in which more than ordinary care is needed, while in accordance with that mistaken humoral pathology so popu lar among the vulgar, the profuse secretion from the irritated glands is regarded as the result of a kind of safety valve arrangement whereby nature seeks to moderate the constitutional excitement attendant upon teething." * * * Page 451: "Not only do nurses attribute to teething the most varied forms of constitutional disturbance, and mothers express serious apprehension as the period of dentition approaches, but medical men hold forth to anxious parents the expectation that their child will have better health when it has cut all its teeth. The time of teething, too, is in reality one of more than ordinary peril to the child, though why it should be is not always rightly understood. It is a time of most active development of the organism—a time of transition from one mode of being to another, in relation to all those important functions by whose due performance the body is nourished and built up. Statistics prove the dangers of this period, and warrant us in regarding the completion of the process of teething as a fair subject for congratulation.

"The error which has been committed with reference to this matter, not merely by the vulgar, but by members of our own profession also, consists, not in over-rating the hazards of the time when changes so important are being accomplished, but in regarding *only one of the manifestations* (though that, indeed, is the most striking one) of the many important ends which nature is then laboring to bring about. * * * The epoch of dentition is to be looked at just in the same way as that in which we regard the epoch of puberty. Constitutional disturbance is more common, and serious disease more frequent, at those times than at others; but their causes lie deeper than the tooth that irritates the gum that it has not pierced in the one case, or than the uterus which has not yielded the due discharge of blood in the other. You might produce hemorrhage from the uterine vessels in the latter instance, or cut through the gum in the former, with no other effect than that of aggravating the condition of the patient. * * *

"I warn you against looking upon all ailments as symptomatic of the local uneasiness which the child suffers in its mouth. Some persons, indeed, act as if they held this notion to its fullest extent; and following up in practice this coarse, mechanical theory, they lance the gums of every child who has not yet cut all its teeth, almost or altogether irrespective of the nature of the affection from which it suffers. Such a course is nothing better than a piece of empiricism which causes the infant much pain, and is useless or mischievous in a dozen instances for one in which it affords relief. Still less is the gum lancet to be used merely with the view of expediting the process that nature is engaged in. The gradual protrusion of the teeth occasions the slow absorption of the superjacent gum, and for this process the division of the gum by a scalpel is at best but a clumsy substitute."

Eustace Smith, M. D., London, on "Wasting Diseases of Infants and Children," says: "Many children are said always to cut their teeth with diarrhœa. Perhaps, however, dentition in these cases is not so entirely to blame as is commonly supposed. No doubt, during the cutting of the teeth the bowels generally are in a state of irritability, for we know at these periods the follicular apparatus of the intestines is undergoing considerable development. The bowels then are ripe for diarrhœa, there is increased sensitiveness to the ordinary exciting causes of purging, but without the exciting causes diarrhœa is by no means a necessary result of such a condition of the alimentary canal. We find that looseness of the bowels is more common in summer than in winter; that is, when the changes of temperature are rapid and unexpected.

It is no doubt the case that functional derangements are frequent during teething, but it is often unfair to attribute these directly to the irritation of an advancing tooth. Looseness of the bowels has been looked upon by some as a natural method of relief to the system, and fears have been held out of grave troubles which might ensue if the looseness were too suddenly arrested. Such fears are groundless. *A catarrhal condition of the bowels should be cured as quickly as possible, especially during dentition.*." (Also see Smith in Quain's Dictionary of Medicine.)

Dr. John Cheyne (1802), "Essay on Diseases of Children," says that diarrhœa is often seen in cases where there is no swelling of the gums, no salivation, nor any appearance of pain or tenderness about the mouth, in cases where the child is cutting its teeth easily, and even in children three months old, who have no teeth at all.

Henoch, Berlin (1882), "Lectures on Diseases of Children," page 62, says: "Every physician knows that the most varied disorders of infants are attributed to teething, and are therefore neglected or even regarded as salutary. In the opinion of the majority of physicians, however, teething is a physiological process which cannot give rise to any morbid phenomena. But it is questionable whether this decided negation is always justifiable, and, although fully recognizing the services thus rendered in restricting "teething" diseases, I cannot suppress certain doubts regarding the universality of this view. We know that the perforation of the teeth is due to the fact that the growing root of the tooth gradually presses the crown forward and pushes through the alveolus after perforation of the overlying gum, which grows continually thinner from the increasing pressure, Vomiting, diarrhœa, even spasmodic cough, may disappear, and have done so, when one or more teeth have passed through." As to cutting with the lancet, he says: "It is now very generally held that every attempt to facilitate the eruption of the teeth, and thus remove the symptoms due to difficult dentition—so called—is absolutely useless. I have, in earlier years, performed scarification with sufficient frequency to convince myself of its entire inutility, and it even appears to me that the cicatrix formed may increase the difficulties connected with the penetration of the tooth."

Strumpell says: "Nervous disturbances are often referred to dentition. The most frequent symptom of this kind is eclampsia. The attacks are sometimes called 'teething convulsions. Although the laity go too far in ascribing all sorts of nervous disorders to teething, still experienced specialists do recognize the *possibility* of such an origin for many cases."

In Buck's "Handbook of the Medical Science," Dr. William H. Flint, New York, writes as follows: "The modern belief that dentition is not the cause of disorders in children has subserved an extremely useful purpose by calling attention to the fact that comparatively few constitutional disturbances are really dependent upon teething, and in doing away with the erroneous belief that the checking of these disorders would exert a harmful effect upon the natural course of dentition."

J. Lewis Smith, New York, in his book on "Diseases of Infancy and Childhood," says: "The opinion formerly entertained by the profession, and now prevalent in the community, that many infantile maladies arise directly or indirectly from dentition, is erroneous. Still, there are physicians of experience who believe that teething is a common cause of certain maladies, especially of functional derangements, even of organs remote from the mouth. On the other hand, equally good observers, and the number is increasing, almost wholly ignore the pathological results of dentition. They say that, as it is a strictly physiological process, it should, like other processes of the kind, be excluded from the domain of pathology. * * * Every physician is called now and then to cases of serious disease, inflammatory and other, which have been allowed to run on without treatment, in the belief that the symptoms were the result of dentition. I have known acute meningitis, pneumonitis, and entero-colitis, even with medical attendance, to be overlooked, and the symptoms attributed to teething during the very time when appropriate treatment was most urgently demanded. Many lives are annually lost from entero-colitis, the parents and friends believing the diarrhœa to be symptomatic of dentition, a relief to it, and therefore not to be treated. Such mistakes are traceable to the erroneous doctrine once inculcated in the schools, and still held by many of the laity, that dentition is directly or indirectly a common cause of infantile diseases and derangements. * * * It is certain that in most cases of diarrhœa which are attributed to dentition there are other causes, such as unsuitable food, or bad housing or residence in an unsalubrious locality. It is certain, as regards city infants, that the chief causes of diarrhœa during the period of dentition are strictly anti-hygienic, dentition being quite subordinate as a cause, and probably ordinarily not operating at all as such. But when, as sometimes happens, at each period of dental evolution, the infant is affected with diarrhœa, the influence of teething is apparent. Such cases enable us to see that teething may really sustain a causative relation to certain diseases not located in the buccal cavity."

Referring to the lancet, he writes that it is much less frequently employed than formerly. It is used more by the ignorant practitioner, who is deficient in the ability to diagnosticate obscure diseases, than by one of intelligence, who can discern more clearly the true pathological state. "It is well to bear in mind the remark of Trousseau, that the tooth is not released by lancing the gum over the advancing crown. The gum is not rendered tense by pressure of the tooth, as some seem to think, for the incision remains linear, and unites by first intention in a day or two. Thus the effect can often last but a day or more. It may help us to understand how active, how powerful, the process of absorption is, if we reflect that the roots of the first teeth are more or less absorbed by the advancing second set, without much pain or suffering from the pressure. If the calcareous particles of the teeth are so readily absorbed what is the foundation for the belief that the soft tissue of the gum is absorbed with difficulty? Too much importance has evidently been attached to the supposed tension and resistance of the gum in the process of dentition."

Dr. James F. Goodhart, London, "Diseases of Children" (1885): "Dentition is usually held to be the cause of many ailments, but to what extent it is really so is doubtful. The time of dentition is one of transition. A uniform and bland diet is changing for one of greater variety, and febrile attacks, diarrhœa, vomiting, which are so rife at this time, are more satisfactorily explained by indigestibility of food than by some occult influence of tooth-cutting."

Dr. Carl Gerhardt, Jena (1871), in his "Children's Diseases," says: "As in domestic life and among physicians there is at present very often an inexcusable slighting of children's diseases, because of the possibility of their being caused by teething, these are therefore neglected or misinterpreted; hence it is important to know that teething very rarely causes disorder, and the physician should consider the dentition at fault *only after careful examination of the food and nutrition of the child, and after an exact physical examination.* The acceptance of this theory of tooth-irritation does not alter or render unnecessary the usual treatment of the remote disorder. Very often there lies behind the teething faults of nutrition which, misunderstood or ignored, are followed by most serious consequences."

In Gerhardt's book, Vol. I., page 415, Dr. A. Jacobi, of New York, says of summer diarrhœa: "It arises from overfeeding, heat or bad air, never from teething." Dr. Jacobi has also said that "the alleged relation between teething and disease is, to say the least, a very doubtful one. * * * Nothing is more harmful than overfeeding. A child of one to two months should be nursed every two or three hours; of six months and more, say five times in twenty-four hours, and not more. If a child becomes thirsty between nursing-times, give it water or barley water."

I shall be surprised if it be not the belief of most present that teething is the most frequent cause of children's disorders; but it ill becomes us to be inconsiderate of the opinions of a large majority of those who give their whole time to the investigation and treatment of children's diseases, and these opinions are decidedly against such belief. No one claims that teething may not *ıxceptionally* cause disturbanee, but the exception must not be made the rule. I am satisfied that too great laxity may be charged against the profession generally for ignoring other and more potent causes of disorder and ascribing every thing to teething. When the teeth are making their appearance, other changes are going on which require attention. Toward the close of the first year, not before, the pancreas, like the salivary glands, becomes active, so that, before this time, should starchy foods be given, they would doubtless cause indigestion, diarrhœa, or both. What an error it would be now to attribute to teething, as would most likely be done, this disorder of the digestive system. It is often observed that a correction simply of the errors of diet removes the disorder previously charged to teething. Mothers nurse their children too often, as a rule, and too much at a time, and rarely give them water, so that the baby's stomach obtains no rest day nor night. An adult stomach could not tolerate such abuse; it is not remarkable that the child suffers. So I feel sure that the safer ground is that teething may *exceptionally* cause disturbances, *as a rule* none.

DISCUSSION.

The paper elicited considerable spirited discussion.

Dr. Vander Veer thought that teething often caused trouble, and said: When Dr. Russell has carried a child day and night, or been disturbed by another's doing the same, he will be willing enough to believe that teething causes mischief.

Dr. Culver believed, reasoning from analogy, that teething might exceptionally give rise to disturbances, but, as a general rule, to none.

Dr. Ward remarked: No one claims that teething often causes serious disease, as meningitis, as referred to in the paper, but that it is the frequent cause of disorders I have no doubt. Saying that it is a physiological process and therefore incapable of causing pain is as absurd as it would be to say that digestion is never painful because physiological. I think good is often done by lancing the gums. A slight diarrhœa will do no harm to a teething child.

Dr. Thompson spoke vigorously against the idea that teething is a fruitful cause of children's diseases, as generally believed. He thought lancing of the gums useless, if not actually harmful.

Dr. Townsend believed that teething, like phimosis, might and often did give rise to reflex disturbances. The diarrhœa accompanying teething, no boubt in his mind, was frequently due to reflex irritability of the sympathetic vaso-motor system, where, dilatation of intestinal blood-vessels taking place, transudation of serum naturally followed. The same may be said with reference to such dilatation of blood vessels in the brain; here transudation of serum frequently fills the ventricles, with its concomitant results.

Dr. M. J. Lewi could easily believe that teething gave rise to disorders, from the fact that adults suffer so much sometimes when cutting the wisdom teeth.

Dr. Bartlett was of the opinion that only exceptionally was teething a cause of disorder. As he understood the paper, it was not a denial that teething might rarely cause disorder, but a plea for discrimination among causes, and a protest against the too common habit of physicians of siding at once with mothers and nurses in favor of such idea, rather than to examine further for adequate cause.

Dr. Russell, in reply to the criticisms, said: As babies often cry most and are carried most before they begin to cut teeth at all, that argument is not convincing.

It is amusing to see Dr. Ward set up a straw man in order to knock him down. No mention was made in the paper of meningitis as due to teething. He says that teething is fairly comparable with digestion in the matter of pain. Digestion does not cause pain; indigestion causes pain.

It may be said, in reply to Dr. Townsend, that in phimosis there is really what acts as a foreign body causing irritation—a substance that may cause irritation in the adult as well.

As to the pain attending the cutting of the wisdom teeth, it is caused chiefly by biting hard substances against the gum, and this same pain may be experienced when the gum grows over an old root.—*Medical Annals.*

